# New Laboratory Informatics Self-Assessment Tool

**Published:** 2013-08-30

CDC and the Association of Public Health Laboratories have recently released a laboratory informatics self-assessment tool to help state and local public health laboratories assess their own informatics capabilities and gaps across a broad range of topics. The self-assessment tool is the first resource aimed at measuring informatics capabilities in public health laboratories in a systematic and comprehensive manner. However, the tool is not limited to use by informatics experts or by public health laboratories. Most of the identified capabilities and guidance are universal in nature and, consequently, can provide valuable assessment and direction to clinical laboratories. The tool is currently available to the public as a downloadable PDF file at http://www.aphl.org/aphlprograms/lss/laboratory-efficiencies-initiative/pages/informatics.aspx.

